# FGA, a new target of histone acetylation, inhibits apoptosis of granulosa cells in follicles

**DOI:** 10.1186/s40659-025-00623-4

**Published:** 2025-07-02

**Authors:** Yongcai Chen, Ming Fang, Wenmiao Duan, Tiantian Yang, Haidan Fan, Meng Lv, Liuhong Zhang, Yao Jiang, Shuo Li, Nian Li, Jiaqi Li, Xiaolong Yuan

**Affiliations:** 1 State Key Laboratory of Swine and Poultry Breeding Industry, National Engineering Research Center for Breeding Swine Industry, Guangdong Provincial Key Laboratory of Agro-Animal Genomics and Molecular Breeding, College of Animal Science, South China Agricultural University, Guangzhou, 510642 Guangdong China; 2 Centre for Healthy Ageing, Health Futures Institute, Murdoch University, Murdoch, WA 6150 Australia; 3 School of Medical, Molecular and Forensic Sciences, Murdoch University, Murdoch, WA 6149 Australia; 4 National Center of Technology Innovation for Pigs, Chongqing, 402460 China

**Keywords:** H3K9ac, FGA, Chromatin accessibility, Follicles

## Abstract

Granulosa cells (GCs) are the main supporting cells for follicles, and histone acetylation has been reported to regulate follicular development. However, the mechanism of histone acetylation regulating follicular development is still unclear in GCs. In this study, we found that *FGA*, fibrinogen alpha chain, mediated the survival and fate of GCs. Knockdowns of HDAC1 and HDAC3 significantly inhibited the mRNA level of *FGA*, while knockdown of HDAC2 notably decreased the protein level of FGA. Moreover, knockdown of HDAC2 repressed the chromatin accessibility and the enrichment level of H3K9ac at -1350/-1454 bp of *FGA*. In addition, *FGA* promoted GCs proliferation and cycle progression by up-regulating the expressions of *PCNA* and *CCNE1*, whereas it inhibited apoptosis by suppressing the expression of *Caspase*3. In vitro, *FGA* was likely to promote follicular development of pigs. In mice, *FGA* inhibited the apoptosis of GCs and increased the number of corpora lutea, as a result, elevating estradiol levels and advancing the day of pubertal initiation. Both in vitro and in vivo experiments, *FGA* promoted follicular development by up-regulating *PCNA* and *CCNE1*, while inhibited follicular apoptosis by down-regulating *Caspase3* and *Caspase9*. Overall, knockdown of HDAC2 repressed transcription by reducing chromatin accessibility and decreasing H3K9ac binding at the *FGA* promoter. *FGA* inhibited apoptosis of GCs by suppressing the expression of *Caspase3* and promoted follicular development. This study showed that *FGA* is a novel target for histone acetylation to regulate follicular development in mammals.

## Introduction

Granulosa cells (GCs), playing an important role in follicular development, is essential for follicular growth and maintenance [1]. It has been reported that the developmental status of GCs promotes follicular activation and subsequently impacts sexual maturation in mice [2]. Excessive apoptosis of GCs in mammalian follicles results in disrupted follicular maturation and ovulatory dysfunction [3]. Previous study has showed that *SMAD4* activates the transcription of *CYP19A1* to suppress GCs apoptosis and follicular atresia in pigs [4]. E2 regulates relative mRNA abundance of *ERβ* isoforms in human GCs, altering *ERβ* protein levels to promote GCs apoptosis and follicular atresia [5]. Moreover, histone deacetylases have been reported to mediate follicular development by regulating gene expression. Knockdown of HDAC1 inhibits the transcription of *SFFD*, leading to the apoptosis of GCs, which in turn impairs follicular development and delays sexual maturation in pigs [6]. Trichostatin A (TSA), a histone deacetylase inhibitor, that attenuates *NLRP3* to induce follicular damage by downregulating HDAC1 and HDAC2 [7], and then promotes ovulation by upregulating the expression of *FSHβ* in mice [8]. Although the above studies suggest that histone deacetylases play an important role in follicular development, the specific mechanisms by which they regulate GCs apoptosis remain to be further explored.

Fibrinogen is a key protein in coagulation mechanisms that regulates cell-matrix interactions vascular adhesion [9], as well as the proliferation of GCs during follicular remodeling [10]. It has been widely reported that fibrinogen is useful in the construction of artificial ovaries [11], with inhibiting apoptosis of GCs and promoting follicular angiogenesis in mouse [12]. Through its interaction with hyaluronic acid and tissue-type plasminogen activator, fibrinogen indirectly regulates ovulation [13]. These findings indicate that fibrinogen is closely associated with GCs growth, which is critical for follicular development and sexual maturation. Fibrinogen alpha chain (*FGA*) is one of the three polypeptide chains (alpha, beta and gamma) [14] that composes fibrinogen, and it has a variety of cellular and physiological functions [15]. Studies on *FGA* and GCs are limited, and the underlying mechanisms that *FGA* promotes follicular development and sexual maturation has not been clarified.

In this study, we found that TSA significantly inhibited *FGA* and apoptosis genes like *Caspase3* and *Caspase9*. Next, we explored how histone acetylation regulated *FGA* transcription and how *FGA* mediated the apoptosis of GCs to influence follicular development and sexual maturation. These works may provide new insights into the mechanisms of follicular development and mammalian sexual maturation.

## Materials and methods

### Cell culture

The human ovarian GCs (KGN) (hereinafter referred to as the “GCs”) used in this study were purchased from Wuhan Pricella Biotechnology Co., Ltd. GCs were cultured with Dulbecco’s modified Eagle’s medium (DMEM, Hyclone, Logan, UT, USA) with 10% FBS (Hyclone, Logan, UT, USA) and incubated at 37 ℃ under 5% CO_2_.

### Transient transfection

Transfection was performed when the GCs at high confluence (> 80%). GCs were transfected with transient transfections of plasmids and oligonucleotides using Lipofectamine™ 3000 Reagent (Thermo Scientific, Waltham, USA), when the cell confluence reaches 70–80%. The oligonucleotides were synthesized by Guangzhou Ruibo (Guangzhou, China) and the sequences are shown in Table 1. The RT-qPCR, Western Blot, EdU and flow cytometry assays were performed at 48 h after cell transfection.

**Table 1:** List of oligonucleotides used in this study

Name	Sequences(5’-3’)
si-HDAC1	AGGCGGTGGTTACACCATT
si-HDAC2	AACCGACAACAGACTGATA
si-HDAC3	GTGGTTATACTGTCCGAAA
si-FGA	GCTGCAGGATGAAAGGGTT

### Animal experiment

Three-week-old C57BL/6 J mice (26 females) from the Laboratory Animal Center of Southern Medical University (Guangzhou, China) were assigned to 4 groups: LV-NC (*n* = 5), LV-FGA (*n* = 8), sh-NC (*n* = 5), and sh-FGA (*n* = 8) group, randomly. The mice were acclimatized and fed for one week at the Laboratory Animal Center of South China Agricultural University, and were injected with the lentivirus of *FGA* by intraperitoneal injection once a week for three weeks, weighed daily and observed for estrus and recorded. The lentivirus was purchased from Guangzhou Dongze (Guangzhou, China). The mice were euthanized at 42 days of age.

### Isolation and culture of porcine follicles

Porcine ovarian tissues collected from a slaughterhouse in Guangzhou were preserved in pre-cooled PBS (containing 1% penicillin-streptomycin) and quickly brought back to the laboratory, washed with PBS until the liquid was clear and free of blood and water, and then transferred to the sterile laboratory, where follicles of 3–5 mm were isolated (excess connective tissue was removed) under the ultra-clean table, washed twice with DMEM/F12 (Biosharp, Beijing, China) medium. 16 follicles were divided into four groups: LV-FGA (*n* = 4), LV-NC (*n* = 4), sh-NC (*n* = 4), and sh-FGA (*n* = 4). Each follicle was individually placed into a well of the 24-well plates containing follicle culture medium and incubated at 38.5 °C with 5% CO_2_ for culture. After 24 h, lentiviral treatment of the follicles were performed and photographed for observation on days 1, 3 and 5, respectively.

### Reverse transcription-quantitative PCR

Total RNA from tissues and cells was extracted following the instructions provided in the RNAfast200 Total RNA Extraction Kit manual (Feijie, Shanghai, China). The purity and integrity of total RNA were detected using UV spectrophotometer (Thermo Fisher Scientific, Waltham, MA, USA). And the extracted RNA was reversely transcribed into cDNA with the PrimeScript RT master MIX (TaKaRa, Tokyo, Japan). Hieff^®^ qPCR SYBR Green Master Mix (2×) (YEASEN, Shanghai, China) and CFX96 Touch Real-Time PCR system (Bio-Rad, Berkeley, CA, USA) was used to quantify the relative expression levels of gene mRNAs. Using the mRNA expression level of GAPDH as internal reference, the relative expression levels of genes were calculated with the 2^−ΔΔCΤ^ method. All primers for RT-qPCR are listed in Tables 2, 3 and 4. 

**Table 2:** Primers used for qPCR in humans

Gene name	Primer sequences (5’-3’)	Product size (bp)
FGA	F: TAGCTGAATTCCCTTCCCGTG R: CTTCCCCAAAGGAGAAGTGTGG	218 bp
HDAC1	F: GCTCCATCCGTCCAGATAACAT R: GCCACAGAACCACCAGTAGACAA	127 bp
HDAC2	F: TTACCTCATGCACCTGGTGTCC R: GACCTCCTTCTCCTTCATCCTCA	175 bp
HDAC3	F: CGGTGTCCTTCCACAAATACG R: CTGGTCATCAATGCCATCCC	125 bp
Caspase3	F: GGAAGCGAATCAATGGACTCTGG R: GCATCGACATCTGTACCAGACC	146 bp
Caspase7	F: CGGAACAGACAAAGATGCCGAG R: AGGCGGCATTTGTATGGTCCTC	143 bp
PLCγ1	F: CATCACGCACTACCAGCAGGTG R: GACGCGCATTAGCATGTGCTCA	151 bp
Bcl-2	F: ATCGCCCTGTGGATGACTGAGT R: GCCAGGAGAAATCAAACAGAGGC	127 bp
BIM	F: CAAGAGTTGCGGCGTATTGGAG R: ACACCAGGCGGACAATGTAACG	136 bp
Caspase8	F: GCTGACTTTCTGCTGGGGAT R: GACATCGCTCTCTCAGGCTC	112 bp
P53	F: CCTCAGCATCTTATCCGAGTGG R: TGGATGGTGGTACAGTCAGAGC	128 bp
Caspase9	F: GTTTGAGGACCTTCGACCAGCT R: CAACGTACCAGGAGCCACTCTT	129 bp
BAD	F: CCAACCTCTGGGCAGCACAGC R: TTTGCCGCATCTGCGTTGCTGT	126 bp
BID	F: TGGGACACTGTGAACCAGGAGT R: GAGGAAGCCAAACACCAGTAGG	125 bp
BAX	F: TCAGGATGCGTCCACCAAGAAG R: TGTGTCCACGGCGGCAATCATC	103 bp
CREB1	F: GACCACTGATGGACAGCAGATC R: GAGGATGCCATAACAACTCCAGG	135 bp
GSK3β	F: CCGACTAACACCACTGGAAGCT R: AGGATGGTAGCCAGAGGTGGAT	150 bp
STAR	F: TACGTGGCTACTCAGCATCGAC R: TCAACACCTGGCTTCAGAGGCA	142 bp
PCNA	F: CAAGTAATGTCGATAAAGAGGAGG R: GTGTCACCGTTGAAGAGAGTGG	126 bp
IKBA	F: TCCACTCCATCCTGAAGGCTAC R: CAAGGACACCAAAAGCTCCACG	101 bp
SP1	F: ACGCTTCACACGTTCGGATGAG R: TGACAGGTGGTCACTCCTCATG	112 bp
MCL1	F: CCAAGAAAGCTGCATCGAACCAT R: CAGCACATTCCTGATGCCACCT	151 bp
MYC	F: CCTGGTGCTCCATGAGGAGAC R: CAGACTCTGACCTTTTGCCAGG	128 bp
CCND1	F: TCTACACCGACAACTCCATCCG R: TCTGGCATTTTGGAGAGGAAGTG	133 bp
CDK7	F: GCACACCAACTGAGGAACAGTG R: AAGTCGTCTCCTGCTGCACTGA	115 bp
CDK2	F: ATGGATGCCTCTGCTCTCACTG R: CCCGATGAGAATGGCAGAAAGC	97 bp
CCNE2	F: CTTACGTCACTGATGGTGCTTGC R: CTTGGAGAAAGAGATTTAGCCAGG	126 bp
CCNH	F: CGATGTCATTCTGCTGAGCTTGC R: TCTACCAGGTCGTCATCAGTCC	128 bp
CCNE1	F: TGTGTCCTGGATGTTGACTGCC R: CTCTATGTCGCACCACTGATACC	123 bp
CDK4	F: CCATCAGCACAGTTCGTGAGGT R: TCAGTTCGGGATGTGGCACAGA	103 bp
GAPDH	F: ATGGGGAAGGTGAAGGTCGG R: CCTGGAAGATGGTGATGGGATT	181 bp

**Table 3:** Primers used for qPCR in pigs

Gene name	Primer sequences (5’-3’)	Product size (bp)
FGA	F: AAGAGCGAGGTCCACACAAG R: CACCGCAGTCAGAACCATCT	205 bp
Caspase3	F: GGATTGAGACGGACAGTGGG R: CCGTCCTTTGAATTTCGCCA	124 bp
Caspase8	F: CTCTGCCTACAGGGTCATGC R: AGGATGGCCCTCTTCTCCAT	162 bp
Caspase9	F: AACTTCTGCCATGAGTCGGG R: CTGGCCTTGGCAGTCAGG	128 bp
P53	F: ACGCTTCGAGATGTTCCGAG R: TTTTATGGCGGGAGGGAGAC	137 bp
BAX	F: GACAGGGGCCCTTTTGCTTC R: CCGCCACTCGGAAAAAGACT	227 bp
BAK	F: CTCGTCCACATCAGAGGAGC R: CTAGGTTCTAGGGGCAGGGT	147 bp
BIM	F: GAGCGGCAAGCTTCCATGAG R: AAGAAAACAGCATTACCCTCCTTG	130 bp
Bcl-2	F: GGATAACGGAGGCTGGGATG R: CACTTATGGCCCAGATAGGCA	150 bp
MCL1	F: GAAGGCGTTAGAGACCCTGC R: TGCCCCAGTTTGTTACTCCG	167 bp
BAD	F: AGTCGCCACTGCTCTTACCC R: TCTTGAAGGAACCCTGGAAATC	172 bp
PCNA	F: GCAGAGCATGGACTCGTCTC R: TTGGACATGCTGGTGAGGTT	120 bp
CDK1	F: AAGTGTGGCCAGAAGTGGAG R: CCAGAAATTCGCTTGGCAGG	157 bp
CDK2	F: TTTGCTGAGATGGTGACCCG R: GCTGAAATCCGCTTGTTGGG	254 bp
CDK4	F: GGCCCTCAAGAGCGTAAGAG R: GTCTCTCGATCAGTTCGGGC	165 bp
CCNA2	F: AACTTCAGCTTGTGGGCACT R: AACGAGGTGCTCCATTCTCA	140 bp
CCNB1	F: AGATCGCAGCAGGAGCTTTT R: CCTCGATTCACCACGACGAT	151 bp
CCNB2	F: AGCCACCCAGGTAGCTAAGA R: GAGAAGGACCCTTTGGAGCC	143 bp
CCND1	F: TTCATTTCCAACCCGCCCTC R: AGAAGGGCTTCGATCTGCTC	182 bp
CCND2	F: AAGAGACCATTCCGCTGACG R: TTCTCATTGGGCTGAGGCAG	45 bp
CCNE1	F: GATGCGAAGGAACCTGACAC R: AGGAACAGGGGTTTTCACAGG	187 bp
CCNE2	F: TGACGGTCATCTCCTGGCTA R: CAGCAGCCGCCAGTATTCTA	177 bp
RRM1	F: CTTCAATGCTGGCACCAACC R: TGTTTCCACCCTGATCCACG	260 bp
GAPDH	F: TGAAGGTCGGAGTGAACGGAT R: CCATTTGATGTTGGCGGGAT	251 bp

**Table 4:** Primers used for qPCR in mice

Gene name	Primer sequences (5’-3’)	Productsize (bp)
FGA	F: AAACATTGAGGACCCCAGCTC R: AGTGTCGATACCTCTCGTTGG	190 bp
CREB1	F: CACAGACCACTGATGGACAGCA R: AGGACGCCATAACAACTCCAGG	138 bp
Caspase7	F: CCGTCCACAATGACTGCTCTTG R: CCCGTAAATCAGGTCCTCTTCC	131 bp
Caspase3	F: GGAGTCTGACTGGAAAGCCGAA R: CTTCTGGCAAGCCATCTCCTCA	113 bp
Caspase8	F: CACACTTGGGAGCCAGCGAAAA R: AGCCGTCTCATGCTGGCAGTAT	115 bp
Caspase9	F: GCTGTGTCAAGTTTGCCTACCC R: CCAGAATGCCATCCAAGGTCTC	124 bp
BAX	F: AGGATGCGTCCACCAAGAAGCT R: TCCGTGTCCACGTCAGCAATCA	103 bp
P53	F: TCCTCAAGAGTGATGAAGGTGGC R: ACGGTGGATGTAGTTCTTCCGC	110 bp
Bcl-2	F: CCTGTGGATGACTGAGTACCTG R: AGCCAGGAGAAATCAAACAGAGG	123 bp
BIM	F: GGAGATACGGATTGCACAGGAG R: CTCCATACCAGACGGAAGATAAAG	156 bp
STAR	F: GTGCTTCATCCACTGGCTGGAA R: GTCTGCGATAGGACCTGGTTGA	113 bp
MCL1	F: AGCTTCATCGAACCATTAGCAGAA R: CCTTCTAGGTCCTGTACGTGGA	125 bp
SP1	F: CTCCAGACCATTAACCTCAGTGC R: CACCACCAGATCCATGAAGACC	133 bp
PCNA	F: CAAGTGGAGAGCTTGGCAATGG R: GCAAACGTTAGGTGAACAGGCTC	112 bp
P65	F: TCCTGTTCGAGTCTCCATGCAG R: GGTCTCATAGGTCCTTTTGCGC	150 bp
CDK4	F: CATACCTGGACAAAGCACCTCC R: GAATGTTCTCTGGCTTCAGGTCC	135 bp
CCNB2	F: GCACTACCATCCTTCTCAGGTG R: TGTGCTGCATGACTTCCAGGAC	137 bp
CCNE1	F: AAGCCCTCTGACCATTGTGTCC R: CTAAGCAGCCAACATCCAGGAC	155 bp
GAPDH	F: CCGTATCGGACGCCTGGTTA	126 bp
	R: CCGTGGGTAGAGTCATACTGGAAC	

### EdU cell proliferation assay

The cell proliferation assay was performed by the Cell-LightTM EdU Apollo 567 In Vitro Kit (RiboBio, Guangdong, China). The GCs were treated with 50 µM EdU solution at 37 °C for 2 h, washed twice with PBS, and then fixed with 80% acetone for 30 min. The GCs were washed twice with PBS and permeated with 0.5% Triton X-100 for 10 min, and then incubated with 1× Apollo for 30 min. The GCs were incubated with Hoechst for 30 min. Three independent replicates were set up for each treatment group, and three fields of view from each replicate were randomly selected for observation by inverted Nikon ECLIPSE Ti2 fluorescence microscope (Nikon, Tokyo, Japan). Cell proliferation rate was calculated as the number of EdU-stained cells (red) compared with the number of Hoechst-stained cells (blue).

### Flow cytometry assay

The apoptosis rate of cells was detected by Annexin V-FITC Apoptosis Detection Kit (BioVision, Milpitas, CA, USA). The cells were collected and washed twice with PBS, and 500 µL 1× AnnexinV Buffer was added to gently suspend the cells. Subsequently, 5 µL Annexin V-FITC and 5 µL PI staining solution were added to cells, and gently mixed. The cells incubated with Annexin V-FITC and PI staining solution for 15 min in darkness. The cell cycle distribution of GCs was detected by Cell Cycle Detection Kit (KeyGEN, Jiangsu, China). The cells were collected and washed with PBS. 500 µL PI/RNase Staining Buffer was added into each tube of cells sample. Cells were re-suspended slowly and incubated with PI/RNase Staining Buffer for 15 min at 37 °C in darkness. Finally, cell apoptosis rate and cycle distribution were analyzed via flow cytometry (BD, USA) and Flowjo software.

### Western blot assay and antibodies

Cells and tissues were lysed using RIPA (Bestbio, Shanghai, China) containing 1% Protease Inhibitors (Biosharp, Beijing, China) to obtain protein samples. The total protein concentration was measured using the BCA Protein Assay Kit (Biosharp, Beijing, China). Protein separation was performed by electrophoresis using Future PAGETM protein precast gels. The separated proteins were transferred to a polyvinylidene fluoride (PVDF) membrane using the eBlot™ L1 membrane converter (GenScript, Nanjing, China). The PVDF membranes were blocked with 5% skim milk powder for 2 h, followed by incubation with the primary antibody solution at 4 °C for 12–16 h. The primary antibodies used were anti-FGA (20645-1-AP, Proteintech, 1:1000), anti-GSK-3β (4198-1-AP, Proteintech, 1:1000), anti-MCL1 (38113, Signalway, 1:1000), anti-PCNA (60097-1-Ig, Proteintech, 1:10,000), anti-STAR (bs-20387R, Bioss, 1:1000), anti-BIM (21280-1, Signalway, 1:1000), anti-CCNE1 (AF4713, Affinity, 1:1000), anti-CCNE1 (AF4713, Affinity, 1:1000), anti-CCND1 (AF0931, Affinity, 1:1000), anti-Caspase3 (AF6311, Affinity, 1:1000), anti-Caspase8 (AF6442, Affinity, 1:1000), anti-Caspase9 (AF6348, Affinity, 1:1000), anti-α-Tubulin (10068-1-Ap, Proteintech, 1:1000), anti-GAPDH (YN5585, Immunoway, 1:5000), anti-P53 (10442-1-AP, Proteintech, 1:1000), anti-CDK4 (DF6102, Affinity, 1:1000) and anti-P65 (10745-1-AP, Proteintech, 1:1000). The horseradish peroxidase-conjugated goat anti-rabbit immunoglobulin G (IgG) (ab205718, abcam, 1:5000) and goat anti-mouse IgG (L3032-1, Signalway, 1:20,000) were used as the secondary antibodies. The PVDF membranes were incubated in the secondary antibody dilution for 1.5–2 h at room temperature. ECL development solution was added dropwise to the PVDF membrane, and on-line development was performed using a fully automated chemiluminescence image analysis system.

### HE staining

The number of follicles and corpus lutea were detected by HE Staining (Servicebio, Wuhan, China). The largest cross section cut along the suspensory ligament of the mouse ovary was stained with hematoxylin-eosin, and images were observed and acquired under a Nikon ECLIPSE Ti2 microscope (Nikon, Tokyo, Japan).

### Enzyme-linked immunosorbent assay

The concentration of E2 in mouse serum was detected using Mouse E2 ELISA Kit (JM-02849 M2) produced by Jingmei Biotechnology Co (Jiangsu, China). For detailed operation, please refer to the instruction manual of the kit. 100 µL of standards of different concentrations and 100 µL of samples were added into the wells of the ELISA plate. Then 100 µL of horseradish peroxidase-modified detection antibody was added to each well and incubated at 37 ℃ for 1 h. 50 µL of enzyme conjugate working solution and TMB substrate solution were added to each well and incubated at 37℃ for 30 min under light protection. Finally, absorbance at 450 nm was measured using a microplate reader.

### Tunel assay

Detection of ovarian follicular apoptosis were uesd by fluorescent Tunel assay (Servicebio, Wuhan, China) on paraffin sections. Mouse ovaries and pig follicles were prepared into paraffin sections. After dehydration, the paraffin sections were repaired with proteinase K (Servicebio, Wuhan, China). The sections were incubated in the dark with Tunel solution for 1 h, processed with anti fluorescein quencher, and observed under a Nikon ECLIPSE Ti2 fluorescence microscope (Nikon, Tokyo, Japan). Image j (NIH, USA) was used for digitization and statistical analysis.

### Chromatin Immunoprecipitation

The ChIP assay was performed according to the MIKX Chromatin Immunoprecipitation (ChIP) Kit (MIKX, Shenzhen, China) instructions. Cells were transfected with NC or si-HDAC2, then subjected to cross-linking, chromatin isolation, lysis, and digestion, followed by immunoprecipitation, IP elution, DNA purification, and recovery. Finally, ChIP-qPCR was performed to assess the role of H3K9ac on the transcriptional regulation of the FGA promoter region. Table 5 lists all primers for ChIP-qPCR.   

**Table 5:** Primers used for ChIP-qPCR

Region name	Primer sequences (5’-3’)	Product size (bp)
FGA-ChIP-P1	F: CACCATTCTACACTTTTCTTCCGTG	149 bp
	R: GCATCATTGCACTCCAGCCT	
FGA-ChIP-P2	F: CAGGCTGGAGTGCAATGATGC	137 bp
	R: AATACTGCTTAGCTGGTCGTGGT	
FGA-ChIP-P3	F: TCTTCATAAAAGCATGGGGTAGC	105 bp
	R: TTCCCTGCTCTATGTTCTTGGAC	
FGA-ChIP-P4	F: TTTGTTCCTGGGGCTGCTG	135 bp
	R: AGGGTTGGGTGATCTGGCTTA	
FGA-ChIP-P5	F: TACCCTCCTATGCTCCTCTTTTCA	137 bp
	R: GCTCAATTCCCCTCAGCTTGT	

### Chromatin accessibility

Performed according to the Epigen TEK EpiQuik™Chromatin Accessibility Assay Kit (Epigentek, USA), cells were transfected with NC or si-HDAC2. Subsequently, buffer and solution preparation, cell processing, cell lysis, chromatin extraction, chromatin digestion, DNA purification, RT-qPCR of samples, and data analysis.

### Co-immunoprecipitation

Performed according to the instruction manual of ACE rProtein A/G Magnetic IP/Co-IP Kit (ACE, Nanjing, China) for the procedure. We started with cell lysis, followed by magnetic bead rinsing, immunoprecipitation, another round of magnetic bead rinsing, and elution.

### Nuclear-cytoplasmic fractionation

Briefly, the cells were washed with PBS and then lysed by adding Lysis Buffer J, followed by incubation on ice for 5 mins. The lysate was transferred to an enzyme-free centrifuge tube and centrifuged for 10 min. The supernatant contained the cytoplasm, and the pellet contained the nucleus, which was stored at -80 °C before subsequent processing.

### Statistical analysis

Statistical analysis was performed using GraphPad Prism (GraphPad Software, USA). At least 3 biological replicates were included for each experimental group. The Shapiro-Wilk test was using to determine the normal distribution, and the Student’s t-test was utilized to compare the significant differences between two groups. All data are shown as mean value ± standard deviation (SD). The *P* value < 0.05 and *P* value < 0.01 were judged as the statistical significance.

## Results

### Histone acetylation inhibits *FGA* to promote apoptosis in GCs

**Fig. 1:**
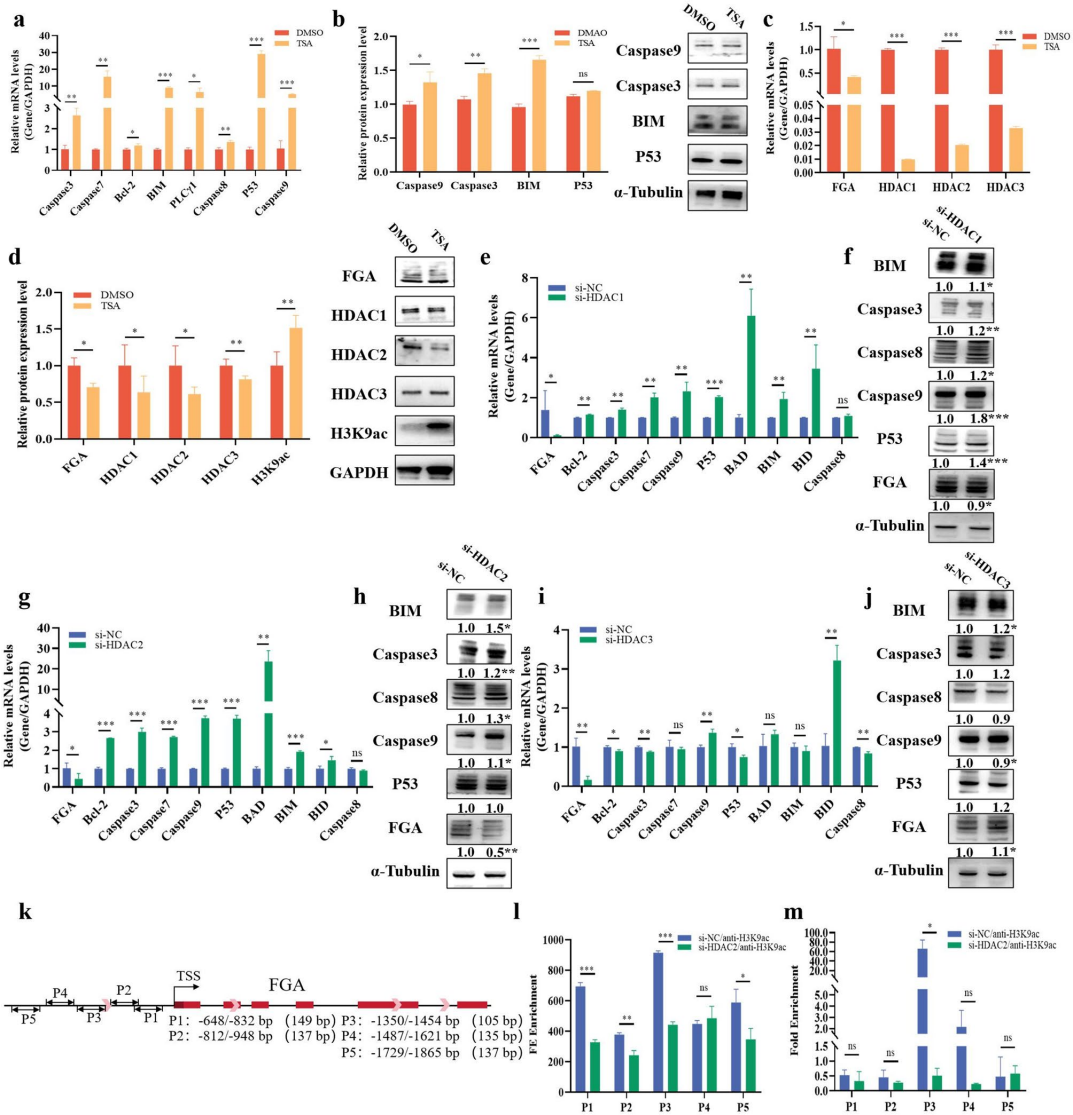
Histone acetylation inhibits *FGA* to promote apoptosis in GCs. Changes in mRNA (**a**) and protein levels (**b**) in apoptosis signaling pathways after TSA treatment. Changes in mRNA (**c**) and protein (**d**) levels of *FGA*, HDACs, and H3K9ac after TSA treatment. The changes in mRNA and protein levels of apoptosis pathway genes and *FGA* after si-HDAC1 (**e**, **f**), si-HDAC2 (**g**, **h**) or si-HDAC3 (**i**, **j**) treatment. segmentation design of *FGA* promoter region (**k**). The level of accessibility modification (**l**) and enrichment (**m**) of H3K9ac on *FGA* promoter (P1, P2, P3, P4 and P5) in cells treated with si-HDAC2 was detected by chromosome accessibility and ChIP-qPCR. In all panels, the statistical significance of differences between means were assessed using Student’s t-test (* *P* < 0.05; ** *P* < 0.01; ns, no significant difference)

TSA, a histone deacetylase inhibitor, inhibits the activities of HDACs by increasing histone acetylation levels, which in turn regulates gene expression [16]. Previously, we treated GCs with TSA and found that the *ACTG1* expression was dose-dependent on the concentration of TSA [17], Based on this, 0.5 µM TSA was chosen for the following experiments in this study. To further explore the specific functions of HDACs in the histone acetylation levels of follicles, GCs were cultured as the cellular model. We found that TSA increased the mRNA and protein levels of cell apoptosis-related genes (e.g., Cysteine aspartate-specific protease family [*Caspase3*] and [*Caspase9*], Tumor suppressor protein p53 [*P53*]) (Fig. 1a, b). TSA promoted the expressions of pro-apoptosis related genes to promote apoptosis of GCs. We then explored whether TSA would alter the level of H3K9ac to inhibit *FGA* expression. The results showed that TSA significantly inhibited the mRNA expression of *FGA* and HDAC1/2/3 (Fig. 1c). The same result was observed at the protein level, with a significantly increase in the modification level of H3K9ac (Fig. 1d). We explored whether HDACs have an effect on apoptosis of GCs, and the results showed that both knockdown of HDAC1 (si-HDAC1) (Fig. 1e, f) and HDAC2 (si-HDAC2) (Fig. 1g, h) were significantly more effective than knockdown of HDAC3 (si-HDAC3) (Fig. 1i, j) in promoting apoptosis pathway genes at both mRNA and protein levels. Knockdown of HDAC1-3 all notably inhibited the mRNA level of *FGA* (Fig. 1e, g, i). The protein level of FGA was noticeably inhibited by si-HDAC1 (Fig. 1f) and si-HDAC2 (Fig. 1h), with si-HDAC2 showing the highest inhibition efficiency. In contrast, si-HDAC3 (Fig. 1j) significantly promoted the protein level of FGA. Based on these results, si-HDAC2 was selected for the following experiments. To explore the HDAC2-mediated-mechanism for the transcriptions of *FGA*, the H3K9ac level of *FGA* promoter, including P1 (-648 to -832 bp), P2 (-812 to -984 bp), P3 (-1350 to -1454 bp), P4 (-1487 to -1621 bp) and P5 (-1729 to -1865 bp) (Fig. 1k), were detected after the treatment of si-HDAC2 in cells. Previous study has shown that H3K9ac regulates gene expression by affecting promoter transcription [18]. The results showed that H3K9ac reduced chromatin accessibility in the P1, P2, P3, and P5 promoter regions of *FGA* (Fig. 1l), along with a significant decrease in H3K9ac enrichment in the P3 region (Fig. 1m). These results suggested that TSA repressed the expression of *FGA* through si-HDAC2-mediated H3K9ac levels and chromatin accessibility in the − 1350/-1454 bp region.

### *FGA* promotes GCs proliferation by accelerating cell cycle progression

**Fig. 2:**
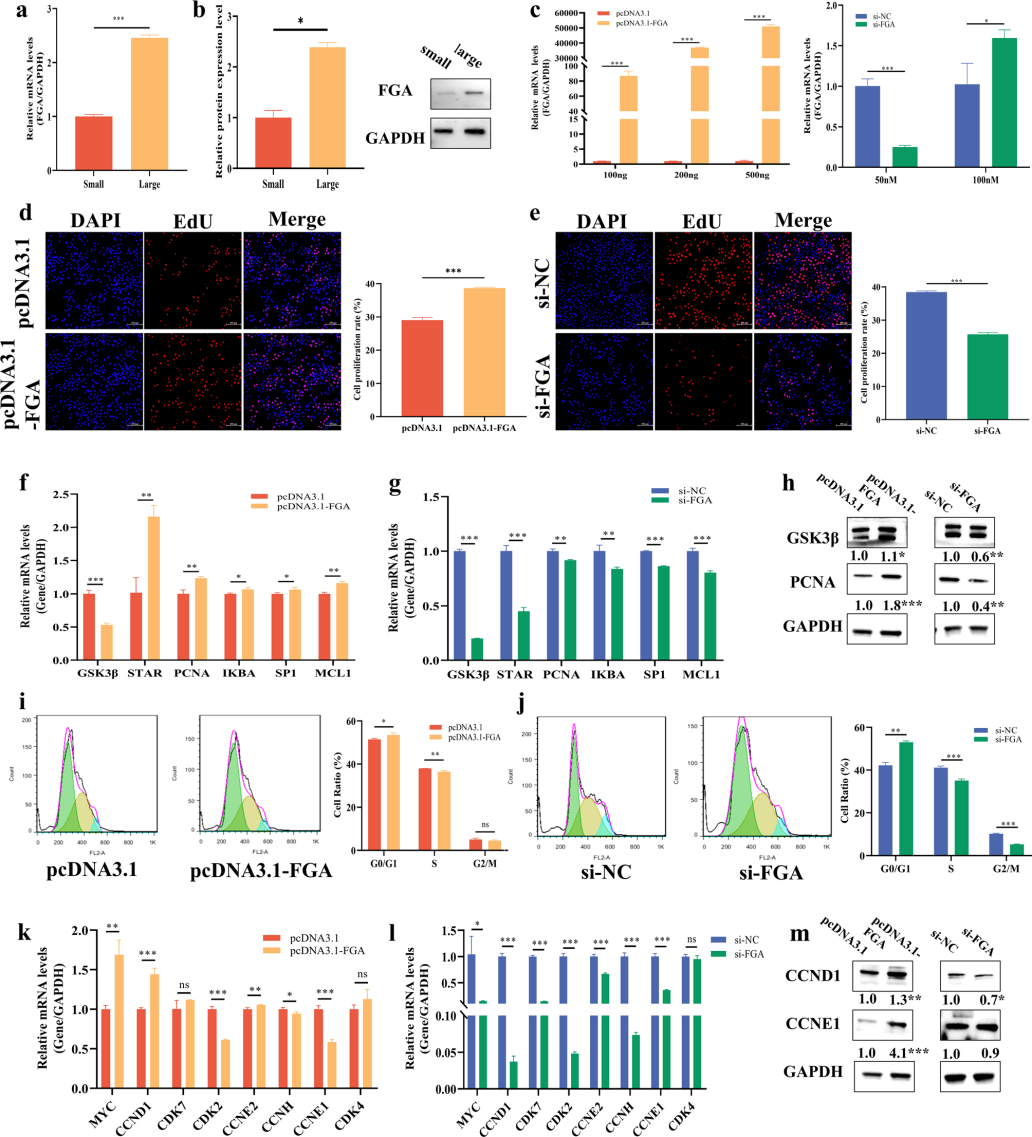
FGA promotes proliferation of ovarian GCs by accelerating cell cycle progression. The mRNA (**a**) and protein (**b**) levels of *FGA* in small (< 3 mm) and large (> 5 mm) follicles. The overexpression and interference efficiency of *FGA* (**c**). The effects of overexpression of *FGA* (**d**) and si-*FGA* (**e**) on the proliferation of cells. The effects of overexpression of *FGA* (**f**) and si-*FGA* (**g**) on the mRNA levels of genes related to cell proliferation. The effects of overexpression of *FGA* and si-*FGA* on the protein levels of genes related to cell proliferation (**h**). The effects of overexpression of *FGA* (**d**) and si-*FGA* (**e**) on the cell cycle. The effects of overexpression of *FGA* (**k**) and si-*FGA* (**l**) on the mRNA levels of genes related to cell cycle. The effects of overexpression of *FGA* and si-*FGA* on the protein levels of genes related to cell cycle (**m**). In all panels, the statistical significance of differences between means were assessed using Student’s t-test (* *P* < 0.05; ** *P* < 0.01; ns, no significant difference)

We found that the expression of *FGA* increased with the expansion of follicular size (Fig. 2a, b) during the follicular growth. To further explore the specific functions of *FGA* in the proliferation of follicles, we constructed the overexpression vector and small interfering RNA of *FGA* (si-*FGA*) (Fig. 2c). The overexpression of *FGA* significantly promoted the proliferation of GCs (Fig. 2d), while the si-*FGA* significantly inhibited the proliferation of GCs (Fig. 2e). The overexpression of *FGA* significantly increased the mRNA expression of cell proliferation related genes (e.g., Steroidogenic Acute Regulatory Protein [*STAR*], Proliferating Cell Nuclear Antigen [*PCNA*], and Specificity Protein 1 [*SP1*]) (Fig. 2f), while si-*FGA* showed opposite results (Fig. 2g). We also found that the overexpression of *FGA* increased the protein levels of GSK3β and PCNA, but si-*FGA* showed opposite effects (Fig. 2h). Meanwhile, we observed a significant increase in the number of GCs in the G0/G1-phase and a significant decrease in the number of GCs in the S-phase after overexpression of *FGA* (Fig. 2i). Conversely, si-*FGA* significantly inhibited the number of GCs entering the S-phases and G2/M-phases (Fig. 2j). The overexpression of *FGA* significantly increased the mRNA expression of cell cycle-related genes (e.g., Myelocytomatosis [*MYC*], G1/S-Specific Cyclin-D1 [*CCND1*]) (Fig. 2k), while si-FGA showed opposite effects (Fig. 2l). We also found that the overexpression of *FGA* increased the protein levels of CCND1 and CCNE1, but si-*FGA* showed opposite results (Fig. 2m). Based on the above results, we concluded that *FGA* promoted GCs proliferation by accelerating cell cycle progression.

### *FGA* inhibits GCs apoptosis by suppressing expression of *Caspase3*

**Fig. 3:**
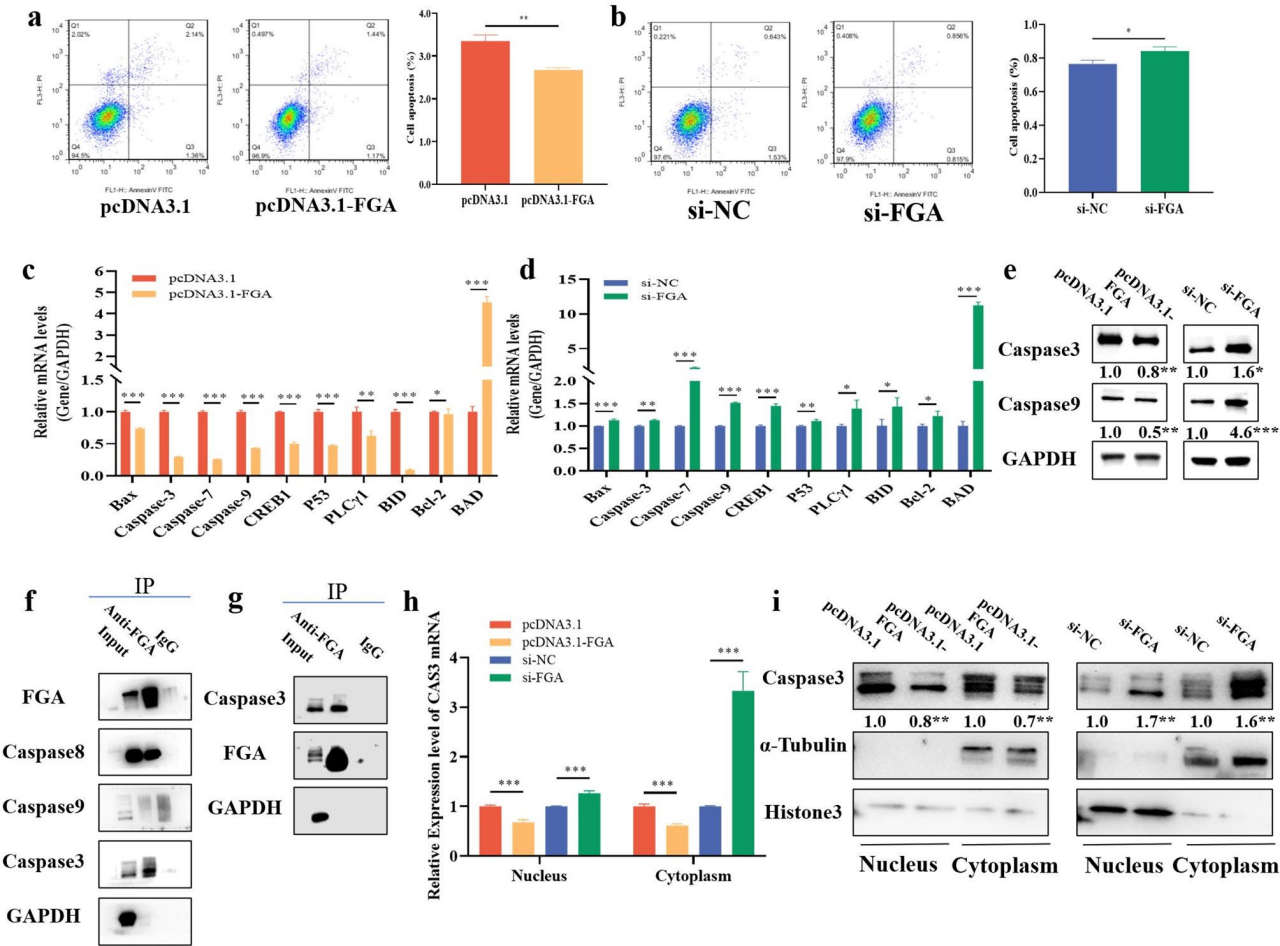
FGA interacts with *Caspase3* to inhibit apoptosis. The effects of overexpression of *FGA* (**a**) and si-*FGA* (**b**) on the apoptosis of cells. The effects of overexpression of *FGA* (**c**) and si-*FGA* (**d**) on the mRNA levels of genes related to cell apoptosis. The effects of overexpression of FGA and si-FGA on the protein levels of genes related to cell apoptosis (**e**). CoIP detected that FGA binds to apoptosis-related proteins (**f**). CoIP reversed validation of apoptosis-related gene protein binding (**g**). *Caspase3* in the nucleus and cytoplasm at the mRNA (**h**) and protein level (**i**). In all panels, the statistical significance of differences between means were assessed using Student’s t-test (* *P* < 0.05; ** *P* < 0.01; ns, no significant difference)

We next investigated the effects of *FGA* on the apoptosis to further determine whether *FGA* inhibited apoptosis in GCs. The apoptosis rate of GCs was significantly decreased by overexpression of *FGA* treatment compared to control (Fig. 3a), whereas si-*FGA* significantly increased the apoptosis rate of GCs (Fig. 3b). The overexpression of *FGA* significantly decreased the mRNA expression of cell apoptosis genes (e.g., BCL2 Associated X Protein [*BAX*], Cysteine aspartate-specific protease family, [*Caspase7*] and [*Caspase9*], *P53*) (Fig. 3c), while si-*FGA* showed opposite effects (Fig. 3d). We also found that the overexpression of FGA decreased the protein levels of Caspase3 and Caspase9, and si-*FGA* showed opposite effects (Fig. 3e). According to the findings of TSA inhibited the expression of *FGA* and apoptosis-related genes (Fig. 1), we tested whether *FGA* inhibited apoptosis by binding to apoptosis-related proteins. Results showed that FGA protein bind to Caspase8, Caspase9, and Caspase3 (Fig. 3f). Subsequently, the reverse validation confirmed that Caspase3 was significantly bound to FGA protein (Fig. 3g). In the nucleoplasmic separation assay, the mRNA and protein levles of Caspase3 were significantly decreased in the nucleus and cytoplasm of GCs after the overexpression of *FGA*, but si-*FGA* showed opposite effects (Fig. 3h, i). In summary, *FGA* inhibited GCs apoptosis by suppressing the expression of pro-apoptotic protein Caspase3.

### *FGA* promotes the growth of porcine follicles

**Fig. 4:**
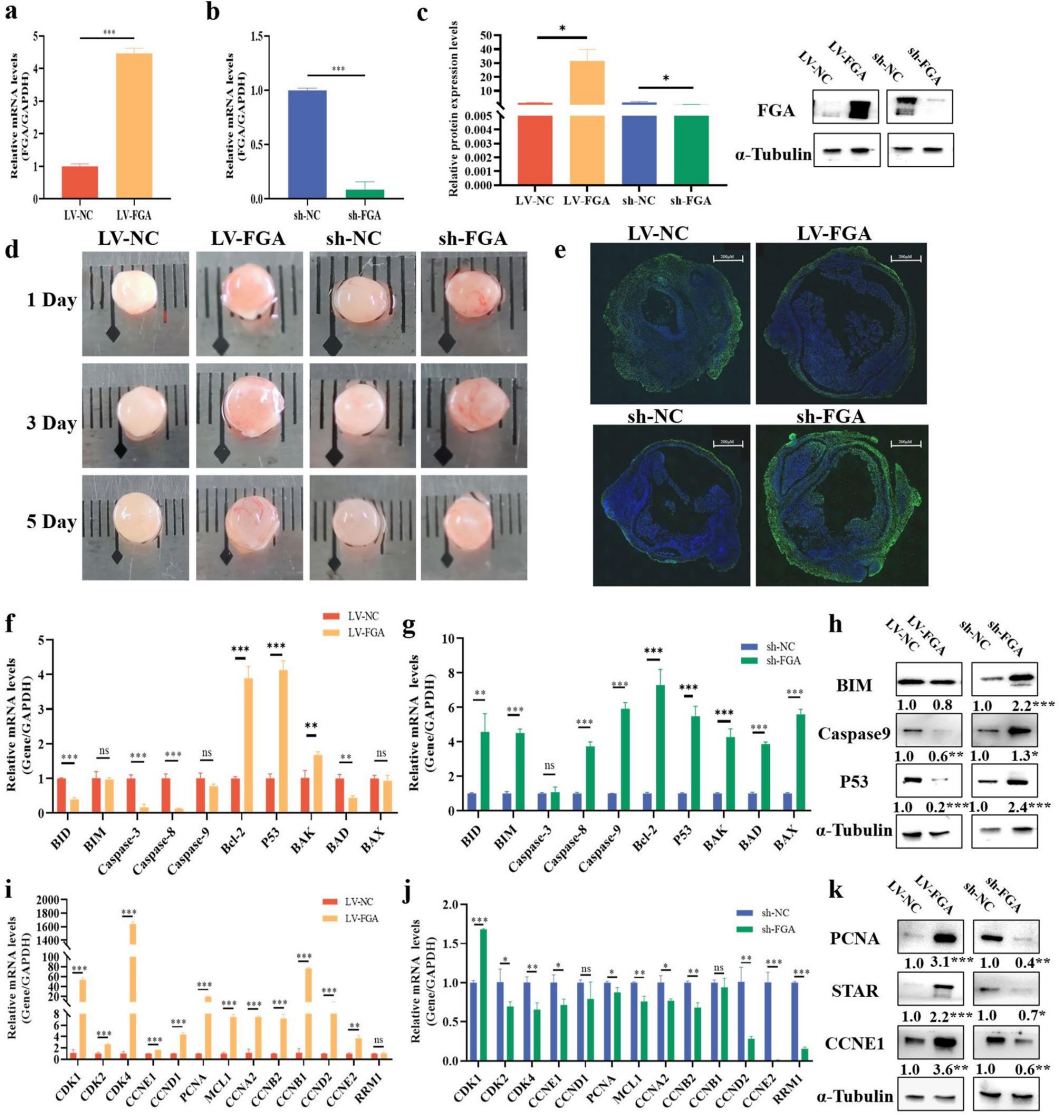
FGA promotes the growth of porcine follicles. Efficiency of lentivirus-mediated FGA overexpression (LV-FGA) (**a**) or FGA knockdown (sh-FGA) (**b**) on the mRNA level. LV-*FGA* and sh-*FGA* efficiency on the protein levels (**c**). The effects of LV-*FGA* and sh-*FGA* on days 1, 3, 5 of porcine follicles (**d**). TUNEL staining of LV-*FGA* and sh-*FGA* treated follicles was performed to assess the apoptotic rates of GCs (**e**). The nuclei stained by DAPI were blue and the positive apoptotic nuclei were green. The scale bar was 200 μm. The effects of LV-*FGA* (**f**) and sh-*FGA* (**g**) on the mRNA levels of genes of cell apoptosis. The effects of LV-FGA and sh-FGA on the protein (**h**) levels of genes of cell apoptosis. The effects of LV-*FGA* (**i**) and and sh-*FGA* (**j**) on the mRNA levels of genes of cell proliferation and cycle. The effects of LV-*FGA* and sh-*FGA* on the protein levels of genes of cell proliferation and cycle (**k**). In all panels, the statistical significance of differences between means was assessed using Student’s t-test (* *P* < 0.05; ** *P* < 0.01; ns, no significant difference)

To investigate whether *FGA* inhibits the apoptosis of GCs and thus promotes follicular growth, the pigs follicles were permeated with the lentiviral-mediated *FGA* overexpression (LV-*FGA*) (Fig. 4a) or lentiviral-mediated *FGA* knockdown (sh-*FGA*) (Fig. 4b). LV-*FGA* promotion and sh-FGA knockdown effects were verified at the protein level (Fig. 4c). Follicles were infected with lentivirus on days 1, 3, and 5 (Fig. 4d). The apoptosis of GCs in follicles was likely decreased by LV-*FGA*, while sh-*FGA* subverted appearances (Fig. 4e). Moreover, LV-*FGA* significantly decreased the mRNA expression of cell apoptosis genes (e.g., *BIM*, *Caspase3*, and *Caspase9*) (Fig. 4f), but significantly elevated in follicles permeated sh-*FGA* (Fig. 4g). LV-*FGA* significantly decreased the protein levels of BIM, Caspase9, and P53, while sh-FGA showed opposite effects (Fig. 4h). Besides, LV-*FGA* significantly increased the mRNA expression of cell proliferation and cycle genes (e.g., *CDK1*, *CDK2*, and *PCNA*) (Fig. 4i), but sh-*FGA* significantly decreased them in follicles (Fig. 4j). Besides, LV-*FGA* significantly increased the protein levels of PCNA, STAR and CCNE1, but sh-*FGA* showed opposite effects (Fig. 4k).

### *FGA* promotes the follicular growth in mice

**Fig. 5:**
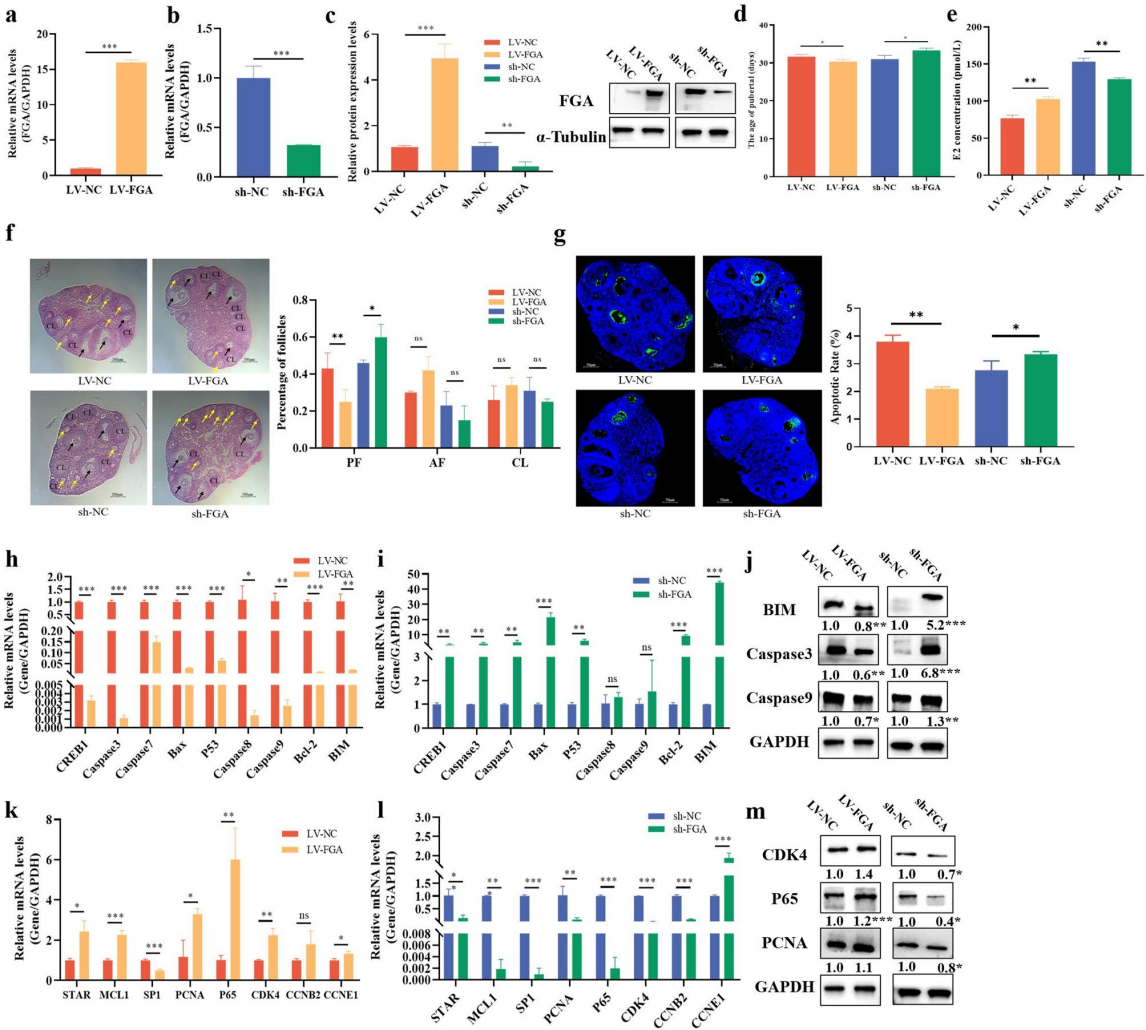
FGA promotes the follicular growth in mice. Efficiency of lentivirus-mediated FGA overexpression (LV-FGA) (**a**) or FGA knockdown (sh-FGA) (**b**) on the mRNA level. LV-*FGA* and sh-*FGA* efficiency on the protein levels (**c**). The effects of LV-*FGA* and sh-*FGA* on the day of pubertal (**d**) and E2 (**e**) levels in mice. The HE (**f**) and TUNEL staining (**g**) of mouse ovary treated by LV-*FGA* and sh-*FGA*. CL represents corpus luteum, black arrows represent antral follicle and yellow arrows represent pre-antral follicles. The scale bar is 500 μm. The effects of LV-*FGA* (**h**) and sh-*FGA* (**i**) on the mRNA levels of cell apoptosis. The effects of LV-*FGA* and sh-*FGA* on the protein (**j**) levels of cell apoptosis. The effects of LV-*FGA* (**k**) and sh-*FGA* (**l**) on the mRNA levels of cell proliferation and cycle. The effects of LV-*FGA* and sh-*FGA* on the protein levels of cell proliferation and cycle (**m**). In all panels, the statistical significance of differences between means was assessed using Student’s t-test (* *P* < 0.05; ** *P* < 0.01; ns, no significant difference)

Based on the above results, *FGA* inhibited apoptosis of GCs and thus promoted follicular development. We next infected lentiviral-mediated LV-*FGA* and sh-*FGA* into the ovaries of 3-week-old C57BL/6 mice (Fig. 5a, b,c). The results showed that LV-*FGA* advanced the day of pubertal initiation, while sh-*FGA* delayed the day of pubertal initiation of mice (Fig. 5d). Moreover, the level of estradiol (E2) in mice serum was significantly upregulated by LV-*FGA*, while sh-*FGA* inhibited the level of E2 in mice serum (Fig. 5e). Compared to the control group, the ovaries of mice with LV-*FGA* showed fewer pre-antral follicles, but more antral follicles and corpora lutea, while sh-*FGA* showed opposite effects (Fig. 5f). These results suggested that *FGA* promoted ovulation of mice. Compared to the control group, LV-*FGA* significantly decreased the apoptotic rate of GCs in the mice ovaries, and sh-*FGA* significantly increased the apoptotic rate of GCs in mice ovaries (Fig. 5g). LV-*FGA* significantly decreased the mRNA expressions of cell apoptosis genes (e.g., *BIM*, *Caspase3*, and *Caspase9*) (Fig. 5h), while sh-FGA showed opposite effects (Fig. 5i). LV-FGA decreased the protein levels of BIM, Caspase3 and Caspase9, and sh-*FGA* showed opposite effects (Fig. 5j). The LV-*FGA* significantly increased the mRNA expressions of cell proliferation and cycle genes (e.g., *STAR*, *CDK4*, *CCNE1*, and *SP1*) (Fig. 5k), while sh-*FGA* showed opposite effects (Fig. 5l). LV-*FGA* increased the protein levels of CDK4, P65, and PCNA, but sh-*FGA* showed opposite effects (Fig. 5m). These results showed that *FGA* inhibited apoptosis, promoted proliferation and E2 secretion in ovarian GCs, and supported ovarian follicle growth and development, thereby advancing the day of pubertal initiation in mice.

## Discussion

Aberrant epigenetic modifications lead to follicular dysfunction, while histone modifications regulate normal gene expressions during follicular development [19]. Histone acetylations, as a common way of histone modifications, have been reported to be involved in follicular development. One previous study reported that histone acetylation regulated the expression of aromatase genes and E2 production in buffalo GCs [16], and sexually mature females showed higher E2 concentrations than sexually immature [20]. How histone acetylation promotes follicular development and sexual maturity is not yet understood. TSA is one of the most widely used histone deacetylase inhibitors [21] and has been reported to increase cellular acetylation levels [22]. Our previous study found that TSA increased the level of H3K4ac to promote *TRLA* transcription in GCs of pigs [17]. In this study, GCs were treated with TSA (Fig. 1a, b) or si-HDACs (Fig. 1e - j). The results showed apoptotic genes including Caspase3, Caspase9, and P53 were significantly up-regulated at both mRNA and protein levels, which were consistent with previous studies that TSA promoted the apoptosis of GCs in Cervus nippon [23], and HDAC inhibitors promoted cell apoptosis [24]. The protein expression of *FGA* following si-HDAC3 treatment showed an opposite trend compared to the mRNA levels (Fig. 1i, j), while the protein expression of *FGA* following si-HDAC1 treatment (Fig. 1e, f) was less significant than that observed with si-HDAC2 (Fig. 1g, h). To explore the mechanism by which HDAC2 inhibited the expression of *FGA*, we analyzed histone acetylation of *FGA* promoter, and found that chromatin accessibility was significantly reduced in the − 648/-832 bp and − 1350/1454 bp of the *FGA* (Fig. 1l), and the level of H3K9ac enrichment at -1350/-1454 bp was significantly suppressed (Fig. 1m). H3K9ac is the marker of transcriptional activation and is regulated by HDAC2 [25]. Si-HDAC2 reverses corticosterone-induced H3K9ac levels of *TGFβR1* [26]. However, site-specific mutagenesis and CRISPR-dCas9-based acetylation techniques might provide higher precision for investigating the direct involvement of H3K9ac in *FGA* promoter regions. For example, a targeted demethylation strategy reduced the methylation level of *ZNF154* promoter, consequently upregulating *ZNF154* expression [27]. Additionally, it was shown that the point mutation *FOXO1* of vectors validated their binding to the *CD36* promoter region and *STEAP4* promoter region, thereby affecting the transcriptional activity of these genes [28]. Our results suggested that H3K9ac targetd - 1350/-1454 bp region to regulate *FGA*’s transcription.

*FGA* is a small molecule of fibrinogen that participates in cell proliferation and apoptosis by regulating cell-matrix interactions [29, 30]. We found that *FGA* promoted GC proliferation (Fig. 2d, e) and accelerated its cycle (Fig. 2i, j) with corresponding increasing in mRNA and protein levels of *PCNA*, *CCND1*, and *CCNE1*. GCs are essential for follicular development, and abnormal proliferation or apoptosis of GCs leads to ovarian insufficiency [31–33]. Based on the effects of si-HDAC2 on inhibiting *FGA* and promoting apoptotic genes such as *Caspase3* and *Caspase9* (Fig. 1g, h), we further investigated the regulatory mechanism of *FGA* in apoptosis in GCs. Our results showed that *FGA* inhibited the apoptosis of GCs (Fig. 3a, b) by downregulating the mRNA and protein expressions of Caspase3 and Caspase9. Reduced expression of *Caspase3* slows down the decline of ovarian reserve function in mice [34]. Additionally, we discovered that FGA bound to Caspase3 (Fig. 3f, g) to suppress GCs apoptosis, and expression of *Caspase3* was inhibited by *FGA* in both nucleus and cytoplasm (Fig. 3h, i). Overall, *FGA* inhibit apoptosis in GCs by suppressing *Caspase3*. Other study has shown that *FGA* regulates cell migration, apoptosis, and other related biological functions through the activation of the PI3K/Akt signaling pathway by integrin α5β1 [14]. Additionally, inhibition of *FGA* expression attenuates mitochondrial ROS production and thrombus formation in zebrafish [35].

GCs play an important role in follicular development, and excessive apoptosis delays sexual maturation in mammals [1]. The expression of *FGA* increased with follicular development (Fig. 2a, b), indicating its involvement in follicular growth. The GCs of small follicles are more proliferative and promote follicular growth, whereas the GCs of large follicles are less proliferative and more functional [36]. As the follicle develops, *FGA* promotes angiogenesis, estrogen production and other related functions. Lentivirus was used to overexpress or knockdown the *FGA* in porcine follicles. In the porcine follicular tissue of *FGA* knockdown lentivirus interference, the mRNA levels of *Bcl-2*, *P53* and *BAK* did not show a decrease. This may be due to tissue-specific [37] interference with the lentiviral system, resulting in certain genes not being affected by the knockdown effect. In future experimental designs, the use of a CRISPR-dCas9 system may help mitigate this phenomenon more effectively. In mice, *FGA* increased the number of antral follicles and corpus lutea (Fig. 5f) and similarly inhibited the level of apoptosis in GCs (Fig. 5g). The main characteristic of sexual maturation is a sharp increase in estrogen or E2 levels [38]. The ovary promotes the expression of steroid hormone synthase through the hypothalamic-pituitary-gonadal axis, which facilitates E2 production and follicular development of GCs [39]. *FGA* promoted the day of pubertal initiation (Fig. 5d) in mice and the secretion of E2 (Fig. 5e). These showed that *FGA* helped to promote sexual maturation in mammals. Meanwhile, changes in mRNA and protein levels of apoptosis and proliferation-related genes in follicles and ovaries also indicated that *FGA* promoted follicular development. *FGA* improves fertility by inhibiting apoptosis of GCs and promoting follicular development. In clinical fertility treatment, the regulation of *FGA* might promote ovulation by improving follicular development and enhancing the quality of oocyte.

## Conclusion

Briefly, the si-HDAC2-mediated decrease in H3K9ac levels inhibits *FGA*’s transcription. Moreover, *FGA* binds to *Caspase3* to inhibit apoptosis in GCs, promote GCs proliferation and enhance E2’s secretion, thereby promoting follicular development and sexual maturation. Overall, our findings suggest that *FGA* may improve fertility by inhibiting apoptosis of GCs and promoting follicular development, and these results may provide theoretical support for the development of FGA-targeted drugs and related strategies to improve follicular development.

## Data Availability

All data sets used and/or analyzed during the current study are available from the corresponding author on reasonable request.
